# Indicators describing the tumor lesion aggregation and dissemination and their impact on the prognosis of patients with diffuse large B cell lymphoma receiving chimeric antigen receptor T cell therapy

**DOI:** 10.1002/cam4.6991

**Published:** 2024-03-20

**Authors:** Xiuyong Dang, Ping Li, Aijun Shen, Yan Lu, Zeyv Zhu, Min Zhang, Wenbin Qian, Aibin Liang, Wenjun Zhang

**Affiliations:** ^1^ Department of Hematology, Tongji Hospital Tongji University School of Medicine Shanghai China; ^2^ Department of Medical Imaging, Tongji Hospital Tongji University School of Medicine Shanghai China; ^3^ Department of Hematology, the Second Affiliated Hospital, College of Medicine Zhejiang University Hangzhou Zhejiang China

**Keywords:** chimeric antigen receptor T cell, diffuse large B‐cell lymphoma, prognosis, relative positioning of tumor lesions

## Abstract

**Introduction:**

Chimeric antigen receptor (CAR) T cell therapy has markedly improved the prognosis of patients with diffuse large B‐cell lymphoma (DLBCL). The relative positioning of tumor lesions in lymphoma varies among patients, manifesting as either aggregation (clumped together) or dissemination (spread throughout the body). Prognostic significance of factors indicating the relative positioning of tumor lesions in CAR T cell therapy remains underexplored. For aggregation, prior research proposed the tumor volume surface ratio (TVSR), linking it to prognosis in chemotherapy. Regarding dissemination, indicators such as disease stage or extranodal involvement, commonly used in clinical practice, have not demonstrated prognostic significance in CAR T cell therapy. This study aims to analyze current indicators of tumor aggregation or dissemination and introduce a novel indicator to assess the prognostic value of tumor lesions' relative positioning in DLBCL patients undergoing CAR T cell therapy.

**Methods:**

This retrospective study included 42 patients receiving CAR T cell therapy. Lesion image information was obtained from the last PET/CT scan prior to CAR T cell infusion, including total metabolic tumor volume, total tumor surface, diameter of lymphoma masses, and the sites of tumor lesions. We evaluated TVSR and bulky disease as descriptors of tumor aggregation. We refined existing indicators, stage III&IV and >1 site extranodal involvement, to distill a new indicator, termed ‘extra stage’, to better represent tumor dissemination. The study examined the prognostic significance of tumor aggregation and dissemination.

**Results:**

Our findings indicate that TVSR, while prognostically valuable in chemotherapy, lacks practical prognostic value in CAR T cell therapy. Conversely, bulky disease emerged as an optimal prognostic indicator of tumor aggregation. Both bulky disease and extra stage were associated with poor prognosis and exhibiting synergistic prognostic impact in CAR T cell therapy.

**Conclusions:**

Overall, the relative positioning of tumor lesions significantly influences the prognosis of patients with DLBCL receiving CAR T cell therapy. The ideal scenario involves tumors with minimal dissemination and no aggregation.

## Condensed abstract

1. The relative positioning of tumor lesions is associated with the prognosis of patients with DLBCL receiving CAR T cell therapy.
2. The tumor lesion with limited dissemination and no aggregation is the most ideal.

## 1 INTRODUCTION

Diffuse large B‐cell lymphoma (DLBCL) is a prevalent haematologic malignancy,^1^ some patients are relapsed or refractory after chemotherapy with a median overall survival (OS) of only 6.3 months.^2^ Chimeric antigen receptor (CAR) T cell therapy has markedly improved the prognosis for these patients. However, a subset of patients exhibits suboptimal responses. Unmet clinical needs prompt us to identify additional prognostic factors and investigate their mechanisms.

Positron emission tomography/computed tomography (PET/CT) is an important examination for evaluating treatment efficacy in lymphoma, which also contains plenty of information.^3^, ^4^ Investigations into PET/CT imaging data have identified prognostic indicators, such as maximum standard uptake value (SUVmax) and total metabolic tumor volume (TMTV), in patients receiving CAR T cell therapy.^5^, ^6^, ^7^, ^8^ The study of the relative positioning of tumor lesions offers a novel approach to understanding lymphoma, an aspect yet to be explored in the context of CAR T cell therapy.

The relative positions of tumor lesions, characterized as either aggregation (clumping together) or dissemination (spreading throughout the body), are crucial in understanding lymphoma pathology. Common descriptors such as bulky disease and disease stage, pertaining to the aggregation and dissemination of lymphoma lesions, have yet to be definitively linked to the efficacy of CAR T cell therapy in various studies.^9^, ^10^, ^11^, ^12^ The concept of tumor volume surface ratio (TVSR), defined as the ratio of TMTV to total tumor surface (TTS), has been proposed in prior research. A higher TVSR, indicating more aggregated tumor lesions, correlates with poorer prognosis.^13^ Another study took the ratio of the distance between the two most distant lesions relative to the patient's body surface area to describe lesion dissemination, finding a significant association with prognosis.^14^ These two studies revealed the prognostic value of tumor aggregation and dissemination in patients undergoing chemotherapy, warranting further investigation in the context of CAR T cell therapy.

Here, we delved into the impact of the relative positioning of tumor lesions in CAR T cell therapy, which is represented by aggregation and dissemination in this study. We condensed two indicators reflecting tumor dissemination in the International Prognostic Index (IPI), stage III&IV and >1 site extranodal involvement, as a novel indicator. We analyzed both existing indicators and new indicators describing tumor aggregation and dissemination to investigate their prognostic value in CAR T cell therapy. Based on the significant prognostic disparities among patients with varying tumor aggregation and dissemination, this study highlighted the importance of the relative positioning of tumor lesions and warranted further comprehensive exploration to enhance its clinical application.

## 2 METHODS

### 2.1 Study design and participants

Consecutive patients treated with autologous CD19 CAR T cells, CD20 CAR T cells, and CD19/20 bi‐specific CAR T cells at Tongji Hospital of Tongji University between January 1, 2019 and December 31, 2021 who fulfilled the following criteria were included in this study: patients were diagnosed as DLBCL according to the 2016 World Health Organization classification of hematopoietic and lymphoid tumors and had at least one PET/CT examination between the last chemotherapy (excluding pretreatment chemotherapy) and the CAR T cell infusion. A total of 42 cases were included.

Data collected include age, gender, date of CAR T cell infusion, date of progression, date of death, CAR T cell target, and treatment response. Ann Arbor staging system was applied. Response of subjects after CAR T cell therapy was evaluated using the Lugano criteria. Lesion image information was obtained from the last PET/CT before CAR T cell infusion, including TMTV, TTS, diameter of lymphoma masses, and the sites of tumor lesions. TVSR was calculated by TMTV/TTS, and bulky disease is defined as lymphoma masses with diameter >5.0 cm in PET/CT. To describe the tumor dissemination, we refined existing indicators, stage III&IV and >1 site extranodal involvement, to distill a new indicator named extra stage, which is defined as tumor lesions above and below the diaphragm and >1 site with extranodal involvement. No commercial sponsors were involved in the study.

### 2.2 18F‐FDG PET/CT examination

Biograph 64 PET/CT imager we used was made by Siemens (Germany). 18F‐FDG was provided by Shanghai Atomic Science and Technology, Ltd (China). The radiochemical purity was more than 95%. Patients were fasted more than 6 h before examination, and their blood glucose was controlled under 11.1 mmol/L. Patients received intravenous 3.70–5.55 mbq/kg 18F‐FDG according to their body mass and underwent PET/CT after lying still for 60 min. The patient first underwent CT scan with the following scanning parameters: tube voltage 120 kV, tube current 170 mA; slice thickness 3.0 mm. Then, imaging with PET was performed, and a three‐dimensional acquisition mode with 5–6 beds and 2.5 min/bed was used. Delayed imaging was performed in the case of diagnostic difficulties, which was performed within (120 ± 15) min after injection of 18F‐FDG in 1–2 bed positions with identical scanning parameters. Attenuation correction was performed on the CT data and reconstructed by an iterative method to finally obtain transverse, sagittal, coronal CT, PET, and PET/CT fusion images.

### 2.3 Calculation of TMTV and TTS

The calculation of TMTV and TTS was based on a watertight mesh model reconstructed from triangular surface patches. The raw data for PET/CT consist of 3D voxel data, which are equivalent to a three‐dimensional array or matrix. Each voxel corresponds to a minimum cubic space of the PET/CT resolution and has a corresponding SUV and CT density value. Before calculating TMTV and TTS, lesion segmentation must be performed, which involves the following steps:

1. Threshold segmentation: A binary voxel data set was computed based on a given SUV range (SUV >41% of SUVmax^15^, ^16^, ^17^), where 1 represents the lesion voxel and 0 represents nonlesion voxel, and the resolution of the binary data set was consistent with the PET/CT voxel resolution.
2. Connected region segmentation: The flood‐fill algorithm was used to calculate each connected component of lesion voxels to obtain independent regions representing different lesions.
3. Hole filling: There may be a few zero‐value voxels within a connected lesion region. Therefore, hole filling was needed to modify zero‐value voxels inside a connected region to nonzero values, that is, filling the holes inside the region to form a completely closed area.

After segmentation, the resulting lesion data are still in voxel form. To facilitate the calculation of volume and surface area, a triangular surface mesh is used to approximate the 3D region's surface expressed in voxel form, and the details are smoothed to avoid voxel‐formed jaggedness, resulting in a watertight 3D mesh model. The various parameters of the triangular mesh can be calculated using well‐established methods. In practice, we directly use the vtk Mass Properties function from the open‐source library vtk to calculate the volume and surface area.

### 2.4 CAR T cell therapy

The structure of the CARs used consists of either single‐chain variable fragment (scFV) of an anti‐CD19 antibody, an anti‐CD20 antibody, or an anti‐CD19/20 bispecific antibody, followed by a 4‐1BB costimulatory domain and a CD3ζ T‐cell activation domain.

Eligible DLBCL patients for this therapy had relapsed or were refractory to their previous treatments, including autologous or allogenic hematopoietic stem‐cell transplantation (HSCT). Expression of CD19/CD20 on malignant B cells was confirmed by flow cytometry or immunohistochemistry. Eastern Cooperative Oncology Group (ECOG) Performance Score <2, normal organ function, measurable disease, and a life expectancy of 12 weeks or more were necessary for eligibility, whereas patients with uncontrollable infection and clinically evident neurological lesions were excluded. All patients underwent lymphodepletion chemotherapy with FC regimen (25 mg/m^2^ fludarabine and 300 mg/m^2^ cyclophosphamide daily for 3 days) on days −5, −4 and −3, followed by intravenous infusion of CAR T cells on day 0. Dose level of CAR T cells was 1–3 × 10^6^ CAR‐positive T cells/kg of body weight. Study protocols were approved by the Ethics Committee of Tongji Hospital of Tongji University and conducted in accordance with the principles of the Declaration of Helsinki. All enrolled patients provided written informed consent for the treatment and follow‐up.

### 2.5 Statistical analysis

Statistical analysis was performed using SPSS 23. *p* Value < 0.05 was considered to be statistically significant. Univariate and multivariate analyses were carried out using Cox proportional hazard model to explore the impact of studied indicators on the prognosis of patients. Spearman correlation analysis was used to analyze the correlation between indicators. Receiver operating characteristic (ROC) curve analysis was performed on continuous variables. Progression‐free survival (PFS) and OS were analyzed by the Kaplan–Meier method and compared using the log‐rank test.

## 3 RESULTS

### 3.1 Patient characteristics

**FIGURE 1:**
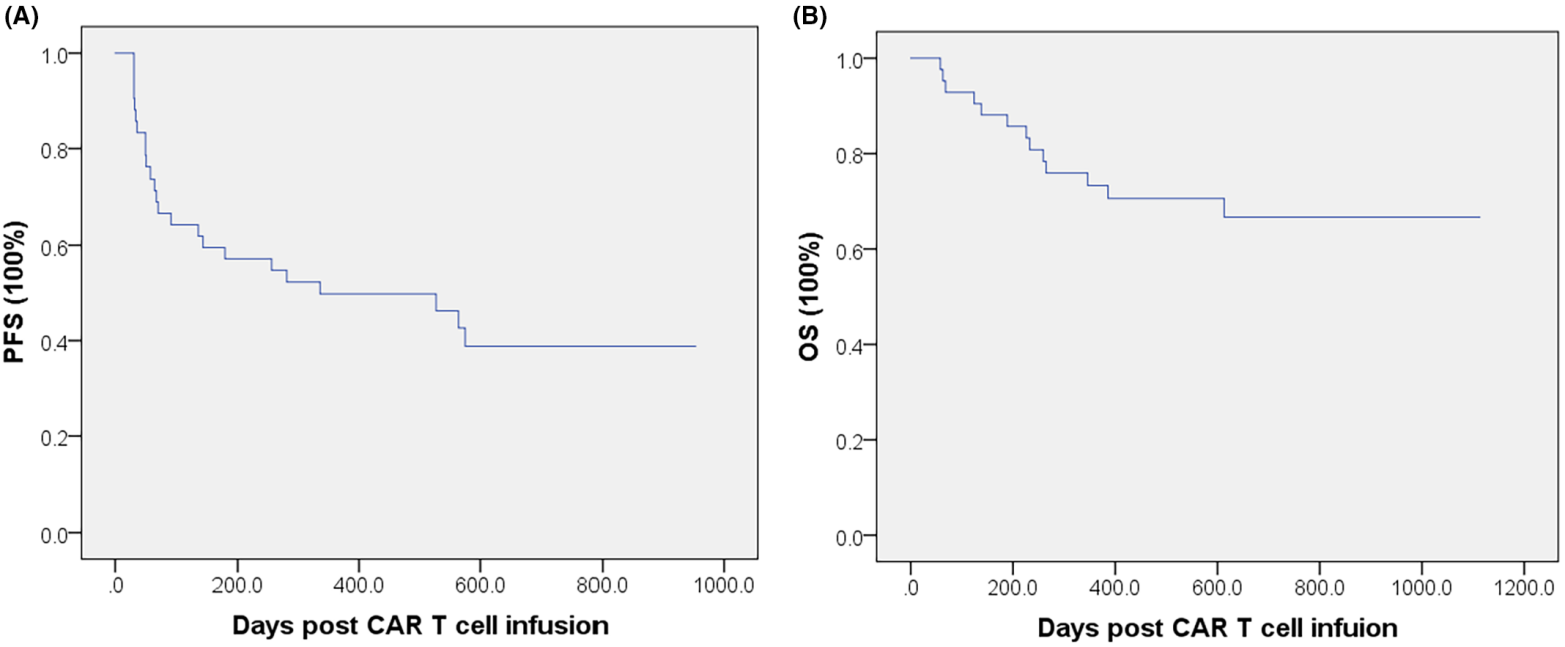
The situation of PFS and OS. (A) Kaplan–Meier curve of all patients based on PFS. Median PFS was 336 days. (B) Kaplan–Meier curve of all patients based on OS

A total of 42 consecutive patients who received CAR T cell therapy were included in this study. Patients' clinical characteristics are described in Table 1. PFS and OS of all patients are shown in Figure 1. The median PFS was 336 days, and 24 (57.1%) patients developed progressive disease (PD) during follow‐up. The media and standard deviation (SD) of TMTV and TTS before CAR T cell infusion were 32,730 ± 141,486 mm^3^ and 6597 ± 21,916 mm^2^, respectively.

**TABLE 1:** Characteristics of patients.

Characteristic	Patients (*n* = 42)
Age	
Median, years (range)	55 (17–75)
>60 years (%)	16 (38.1)
Male (%)	25 (59.5)
CAR T cell target	
CD19 (%)	20 (47.6)
CD20 (%)	8 (19.0)
CD19/20 (%)	14 (33.3)
Ann Arbor stage	
I (%)	4 (9.5)
II (%)	5 (11.9)
III (%)	4 (9.5)
IV (%)	28 (66.7)
Extranodal site (%)	29 (69.0)
Bulky disease >5 cm (%)	11 (26.2)
Patients achieved CR after CAR T cell infusion (%)	25 (59.5)

### 3.2 Describing tumor aggregation, TVSR lacks practical prognostic value, while bulky disease is optimal to predict prognosis in patients receiving CAR T cell therapy

We explored the prognostic value of indicators describing tumor aggregation, which are TVSR and bulky disease.

ROC curve was plotted to determine whether TVSR can predict CR after CAR T cell therapy. The value of TVSR when the maximum of the Youden index is taken is the cutoff value. Predicting CR using TVSR, the AUC is 0.706, *p* = 0.025, cutoff value is 4 mm. Through univariate Cox proportional hazard model, we found that TVSR was prognostic factors for PFS (TVSR > 4 mm: HR = 2.947, *p* = 0.013).

However, we found that TMTV and TVSR were highly correlated with Spearman's rank correlation coefficient of 0.888, *p* = 0.000. In previous study exploring TVSR in patients receiving chemotherapy, TVSR and TMTV were correlated with a lower coefficient (0.50).^13^ As both studies applied a threshold value of 41% of SUVmax to separate the segmented lesion from surrounding space, we compared TMTV and TTS between previous study and our study and found that TMTV and TTS in our study are significantly lower than in previous study (Table 2).

**TABLE 2:** The median ± SD of TMTV and TTS in previous study^13^ and our study.

	TMTV (mm^3^)		TTS (mm^2^)	
	Median	SD	Median	SD
Previous study^13^	279,000	614,000	47,300	91,800
Our study	32,730	141,486	6597	21,916

Compared with chemotherapy, the screening condition of CAR T cell therapy was more stringent, so that the tumor lesion of patients enrolled is more likely to be a few sphere‐like bodies that have similar shapes and lower TMTV and TTS, resulting a stronger correlation between TMTV and TVSR. Given that the calculation of TMTV is more convenient than TVSR, which is TMTV/TTS, TVSR lacks practical prognostic value in CAR T cell therapy.

**FIGURE 2:**
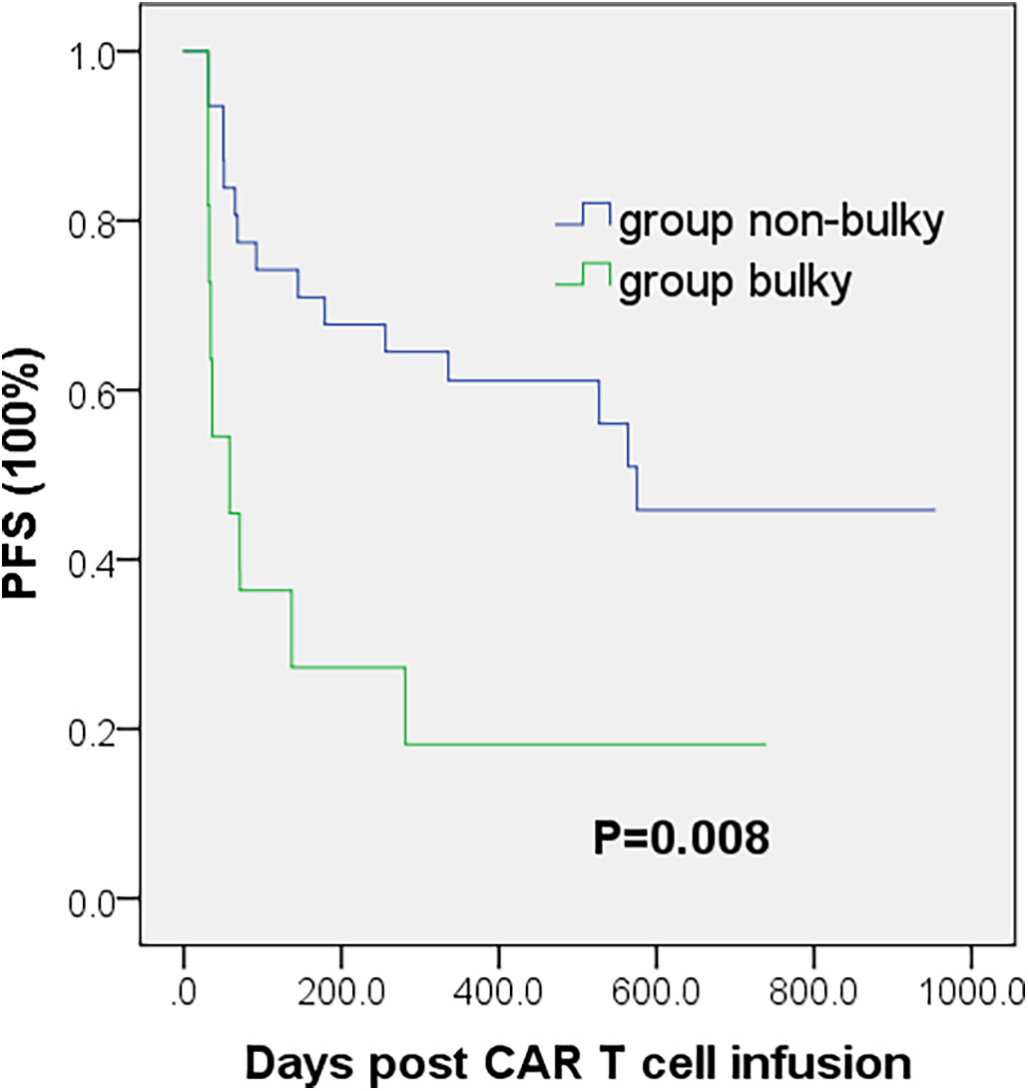
Kaplan–Meier estimates of PFS according to bulky disease.

Based on the bulky disease identified by PET/CT, we divided patients into bulky and nonbulky groups. We plotted Kaplan–Meier curve based on PFS and found that group nonbulky had statistically superior PFS than group bulky (Figure 2).

Spearman's rank correlation coefficient of bulky disease and TMTV are 0.427, *p* = 0.005. Multivariate Cox proportional hazard model including bulky disease and TMTV showed that they are statistically significant for PFS (bulky disease: *p* = 0.036; TMTV: *p* = 0.002). Considering its accessibility and simplicity, we take bulky disease shown in PET/CT as the optimal indicator to describe tumor aggregation.

### 3.3 Describing tumor dissemination, extra stage has prognostic value in patients receiving CAR T cell therapy

We explored the prognostic value of indicators describing tumor dissemination, including indicators from IPI, disease stage III&IV and >1 site with extranodal involvement, and the new indicator we proposed, extra stage.

Univariate Cox proportional hazard model showed that disease stage III&IV and >1 site with extranodal involvement were not prognostic factors in patients receiving CAR T cell therapy (disease stage III&IV for PFS and OS, *p* = 0.864 and *p* = 0.608, respectively; >1 site with extranodal involvement for PFS and OS, *p* = 0.051 and *p* = 0.091, respectively), whereas extra stage, defined as tumor lesions above and below the diaphragm and >1 site with extranodal involvement, is associated with the prognosis (extra stage for PFS, *p* = 0.012; extra stage for OS, *p* = 0.035).

### 3.4 Bulky disease and extra stage have synergistic effect in predicting the prognosis of patients receiving CAR T cell therapy

To comprehensively reflect the relative positioning of tumor lesions, we combined bulky disease and extra stage and explored their ability to stratify patients receiving CAR T cell therapy.

**FIGURE 3:**
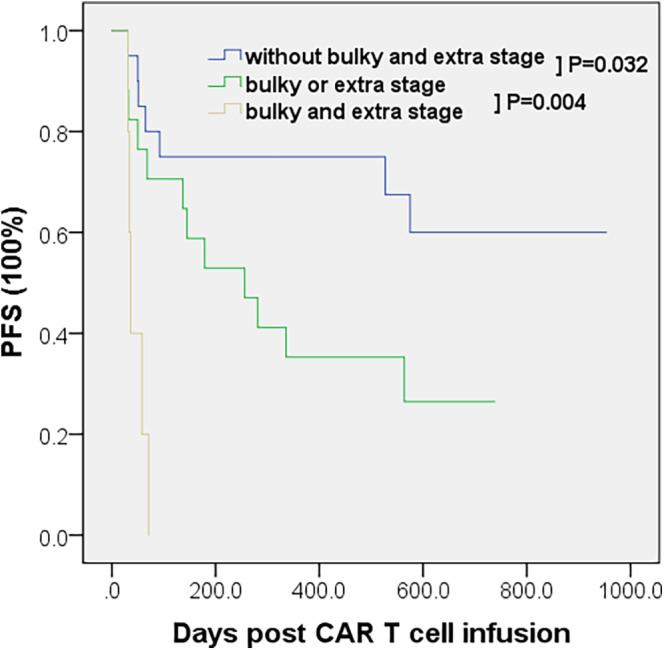
Kaplan–Meier estimates of PFS according to bulky disease combined with extra stage.

Multivariate Cox proportional hazard model including bulky disease and extra stage showed that they are statistically significant for the prognosis. Kaplan–Meier curve based on bulky disease combined with extra stage is plotted (Figure 3). Patients having bulky disease and extra stage had a worse prognosis than patients having only bulky disease or extra stage (*p* = 0.004). Conversely, patients without these characteristics had a better prognosis than those having bulky disease or extra stage (*p* = 0.032), showing the synergistic effect of bulky disease and extra stage.

**FIGURE 4:**
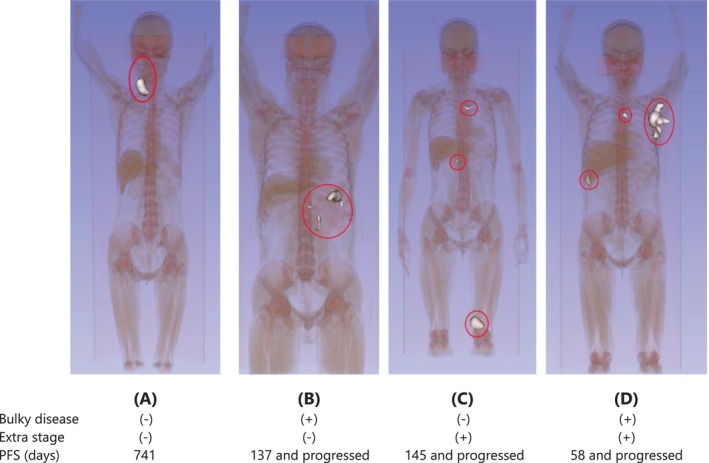
Watertight 3D mesh model of the tumor lesions (marked by red circles) of four patients having the different status of bulky disease and extra stage.

At last, we presented the 3D model of tumor lesions of four patients to visualize our results (Figure 4). Tumor lesions of patient A neither aggregated nor disseminated, indicating better prognosis, whereas patient D, whose tumor lesions are both aggregated and disseminated, has poor prognosis.

## 4 DISCUSSION

This study introduced the aggregation and dissemination of lymphoma lesions and thoroughly explored their prognostic value in patients with DLBCL receiving CAR T cell therapy. Through the analysis of 42 data sets of PET/CT conducted prior to CAR T cell infusion, we obtained TMTV, TTS, diameter of lymphoma masses, and the locations of tumor lesions. TVSR was calculated by TMTV/TTS, as referenced from the aforementioned study. We found that TVSR lacks practical prognostic value in patients undergoing CAR T cell therapy. Conversely, bulky disease shown in PET/CT is optimal to describe tumor aggregation and predict prognosis due to its accessibility and simplicity. We also found the indicator we introduced, extra stage, which reflects the tumor lesion dissemination, is associated with the prognosis.

Our study revealed that TVSR, previously shown to have synergistic prognostic power with TMTV in chemotherapy, is highly correlated with TMTV and does not enhance prognosis prediction in CAR T cell therapy. The main reason is that patients receiving CAR T cell therapy have a relatively lower tumor burden, leading to less heterogeneity in TTS.

Previous studies have noted the adverse effects of bulky disease on the prognosis of patients with lymphoma.^18^, ^19^, ^20^, ^21^ Immunosuppressive tumor microenvironment in the center of the tumor mass and inability of drugs to penetrate bulky disease are the underlying causes of poor prognosis. In this study, bulky disease and TMTV together have a synergistic effect in predicting the prognosis of patients with DLBCL receiving CAR T cell therapy, which underlines the impact of tumor aggregation on the prognosis.

Prior research suggested that the dissemination of lymphoma lesions is associated with poor prognosis.^22^, ^23^, ^24^, ^25^ The absence of adhesion molecules,^22^ abnormality in chemokine receptors and ligands,^23^ and tissue‐specific lymphocyte homing^26^ may be underlying mechanisms driving lymphoma dissemination, suggesting increased activity and drug resistance potential in tumor cells. However, stage III&IV and >1 site extranodal involvement, which both reflect tumor dissemination in IPI, had no prognostic value in CAR T cell therapy. We consider that stage III, tumor lesions above and below diaphragm, signifies the ability to encroach lymph nodes, which cannot be presented by >1 site extranodal involvement. So, we defined tumor lesions above and below diaphragm and >1 site extranodal involvement as extra stage to represent tumor dissemination. Our study showed extra stage had prognostic value in CAR T cell therapy.

Bulky disease and extra stage represent the relative positioning of lymphoma lesions with different aspects, which both indicate poor prognosis. These two indicators can jointly predict the prognosis, suggesting that tumor lesions with limited dissemination and no aggregation are most favorable. Limitations of this study include a small sample size, single center, and retrospective nature. In future studies, we will delve into the mechanisms through which the relative positioning of lymphoma lesions influences prognosis. We will develop more comprehensive, understandable, and accessible indicators to describe the aggregation and dissemination of lymphoma lesions and assess their prognostic value in a larger cohort.

## 5 CONCLUSION

This study suggests that the tumor aggregation described by bulky disease and the tumor dissemination described by extra stage shown in PET/CT prior to CAR T cell infusion are associated with the prognosis of patients with DLBCL undergoing CAR T cell therapy. The combination of these two indicators has a synergistic effect on prognostic prediction. The findings underscore the clinical significance of describing the relative positioning of lymphoma lesions in CAR T cell therapy.

## AUTHOR CONTRIBUTIONS

**Xiuyong Dang:** Conceptualization (lead); methodology (lead); writing – original draft (equal); writing – review and editing (equal). **Ping Li:** Investigation (equal); project administration (lead); resources (supporting). **Aijun Shen:** Data curation (lead); formal analysis (equal); supervision (equal); writing – review and editing (equal). **Yan Lu:** Investigation (supporting); resources (supporting); validation (lead). **Zeyv Zhu:** Conceptualization (supporting); data curation (supporting); formal analysis (supporting); methodology (supporting); project administration (supporting); writing – original draft (supporting). **Min Zhang:** Investigation (supporting); resources (supporting). **Wenbin Qian:** Funding acquisition (equal); supervision (equal). **Aibin Liang:** Conceptualization (supporting); data curation (supporting); funding acquisition (lead); methodology (supporting); resources (supporting); writing – original draft (supporting). **Wenjun Zhang:** Conceptualization (supporting); investigation (equal); methodology (supporting); project administration (equal); resources (equal); writing – review and editing (supporting).

## FUNDING INFORMATION

The study design and data collection of this work was supported by funds from the Ministry of Science and Technology of the People's Republic of China (grant no. 2021YFA1100800 to AL), the National Natural Science Foundation of China (grant nos. 81830004, 81830006, and 82070168 to AL and WQ), Clinical Research Plan of SHDC (grant no. SHDC2020CR6005 to AL), and the Science and Technology Department of Zhejiang Province (grant no. 2021C03117 to WQ).

## CONFLICT OF INTEREST STATEMENT

The authors declare that they have no competing interests.

## ETHICS STATEMENT

Study protocols were approved by the Ethics Committee of Tongji Hospital of Tongji University and conducted in accordance with the principles of the Declaration of Helsinki.

## PATIENT CONSENT STATEMENT

All enrolled patients provided written informed consent for the treatment and follow‐up.

## CLINICAL TRIAL REGISTRATION

Not applicable.

## PERMISSION TO REPRODUCE MATERIAL FROM OTHER SOURCES

Not applicable.

## Data Availability

The data that support the findings of this study are available from the corresponding author upon reasonable request.
