# Correction to “Indicators describing the tumor lesion aggregation and dissemination and their impact on the prognosis of patients with diffuse large B cell lymphoma receiving chimeric antigen receptor T cell therapy”

**DOI:** 10.1002/cam4.70322

**Published:** 2025-01-20

Dang, X., Li, P., Shen, A., Lu, Y., Zhu, Z., Zhang, M., Qian, W., Liang, A., & Zhang, W. (2024). Indicators describing the tumor lesion aggregation and dissemination and their impact on the prognosis of patients with diffuse large B cell lymphoma receiving chimeric antigen receptor T cell therapy. *Cancer Medicine*, 13(6), e6991. doi: 10.1002/cam4.6991.

In the author byline, the spelling of “Zeyu Zhu” has been corrected.

We apologize for this error.

